# Suspected Metronidazole-Induced Acute Pancreatitis in a Three-Year-Old Child Following Treatment for Entamoeba histolytica Gastroenteritis: A Case Report

**DOI:** 10.7759/cureus.113955

**Published:** 2026-08-04

**Authors:** Elsayed M Elsayed, Abdulaziz A Alsuliman, Abdulaziz R Aabeido, Abdul Salam Ghulam, Mariam E Mohamed

**Affiliations:** 1 Pediatrics, Kalba Hospital, Emirates Health Service (EHS), Kalba, ARE; 2 Pediatrics, Fujairah Hospital, Emirates Health Service (EHS), Fujairah, ARE

**Keywords:** acute pancreatitis, drug-induced pancreatitis (dip), entamoeba histolytica infection, metronidazole, pediatric pancreatitis

## Abstract

Acute pancreatitis (AP) is an uncommon but important cause of abdominal pain in children. Pediatric AP has a broad range of etiologies, including biliary disease, infections, trauma, metabolic abnormalities, genetic disorders, and medications. Drug-induced pancreatitis is rare, and metronidazole has been reported as a potential trigger, with most published cases occurring in adults. Reports in children remain exceedingly uncommon. A previously healthy three-year-old boy (weight: 15 kg) developed AP six days after completing a 10-day course of oral metronidazole, 150 mg (10 mg/kg/dose) three times daily prescribed for amoebic gastroenteritis diagnosed by stool microscopy. He presented with severe upper abdominal pain and non-bilious vomiting. Laboratory investigations demonstrated markedly elevated pancreatic enzymes, with a serum amylase level of 3251 IU/L and serum lipase >1,000 U/L. Abdominal ultrasonography revealed mild pancreatic enlargement with edematous changes and a small volume of ascites, consistent with AP. Evaluation for alternative etiologies, including biliary disease, trauma, metabolic abnormalities, systemic illness, and infectious causes (repeat stool microscopy, respiratory viral PCR panel, and urinalysis were negative), did not identify another cause. The patient was managed conservatively with intravenous fluids, analgesia, temporary bowel rest due to persistent vomiting and severe abdominal pain, and gradual reintroduction of oral feeding as tolerated. His symptoms resolved, pancreatic enzyme levels declined markedly during recovery, and follow-up ultrasonography demonstrated complete resolution of the inflammatory changes. This report describes a rare case of suspected metronidazole-associated AP in a young child following treatment for amoebic gastroenteritis. Although a definitive causal relationship cannot be established, the temporal association with metronidazole exposure, together with the systematic exclusion of more common pediatric etiologies, supports the possibility of a drug-related adverse event. Clinicians should consider AP in children presenting with persistent vomiting and upper abdominal pain during or after metronidazole therapy while recognizing that causality cannot be confirmed from a single case report.

## Introduction

Acute pancreatitis (AP) is an increasingly recognized cause of pediatric hospitalization and abdominal pain, with an estimated incidence ranging from three to 13 cases per 100,000 children annually. The diagnosis of AP in children is established when at least two of the following criteria are present: characteristic abdominal pain, serum amylase and lipase levels exceeding three times the upper limit of normal, and imaging findings consistent with pancreatic inflammation [1]. Although most children experience a mild and self-limiting course, early recognition is essential to ensure appropriate supportive management and prevent complications [2].

The etiologies of pediatric AP differ from those observed in adults and include biliary disease, infections, trauma, systemic illnesses, metabolic disorders, genetic predisposition, and medications [2,3]. Infectious causes, particularly viral infections, represent important contributors to pediatric AP, whereas drug-induced pancreatitis (DIP) accounts for only a small proportion of cases [2,3]. Among medications associated with DIP, metronidazole has been reported as a rare potential trigger, with most published cases occurring in adults and limited evidence available in children [4,5].

The diagnosis of DIP remains challenging because there are no universally accepted diagnostic criteria specific to individual medications, including metronidazole. It is primarily considered a diagnosis of exclusion. It is supported by a compatible temporal relationship between drug exposure and symptom onset, fulfillment of established diagnostic criteria for AP, exclusion of alternative etiologies, and consistency with previously reported cases. In some reports, recurrence of pancreatitis following rechallenge with the suspected medication has further supported causality; however, rechallenge is rarely performed in clinical practice because of ethical concerns and the potential risk of harm to the patient.

*Entamoeba histolytica* remains an important cause of gastrointestinal disease worldwide, particularly in developing countries. While intestinal infection and hepatic abscess formation are well-recognized manifestations, pancreatic involvement is exceptionally rare and has been described predominantly in the setting of severe invasive disease [6]. Therefore, distinguishing infection-related pancreatitis from suspected DIP may be challenging when both an underlying infection and its treatment could contribute.

We report a case of suspected metronidazole-associated AP in a previously healthy three-year-old child following treatment for amoebic gastroenteritis. This case highlights the diagnostic challenges in determining the etiology of pediatric pancreatitis. This case emphasizes the importance of a systematic evaluation of alternative causes while maintaining a cautious interpretation of drug-related associations.

## Case presentation

A previously healthy three-year-old boy (weight: 15 kg) presented with a one-day history of watery diarrhea, three episodes of non-bilious, non-projectile vomiting, and mild generalized abdominal pain without fever. On examination, he was alert, hemodynamically stable, and mildly dehydrated, with age-appropriate vital signs. Initial laboratory investigations demonstrated a white blood cell count of 14.89 × 10⁹/L with neutrophilia (absolute neutrophil count 10.0 × 10⁹/L), mild thrombocytosis (479 × 10⁹/L), hypoglycemia (random blood glucose 3.1 mmol/L), hypochloremia (94 mmol/L), and metabolic acidosis (bicarbonate 15.3 mmol/L, venous pH 7.33), while renal and liver function tests were within normal limits. Stool microscopy identified *Entamoeba histolytica* cysts, and stool viral antigen testing for rotavirus and adenovirus was negative, confirming a diagnosis of amoebic gastroenteritis. Baseline serum amylase obtained during his admission was within the normal pediatric reference range (<36 U/L), with no clinical or biochemical evidence of AP. The complete laboratory investigations are summarized in Table 1. Age-specific pediatric reference intervals were interpreted according to the Canadian Laboratory Initiative on Pediatric Reference Intervals (CALIPER) database.

**Table 1:** Laboratory investigations during the first admission ALT: alanine aminotransferase, AST: aspartate aminotransferase

Investigation	Result	Pediatric reference interval
Complete blood count		
White blood cell count (×10⁹/L)	14.89	5.0-17.0
Absolute neutrophil count (×10⁹/L)	10.0	1.5-8.5
Absolute lymphocyte count (×10⁹/L)	1.55	2.0-8.0
Hemoglobin (g/dL)	12.4	11.5-13.5
Mean corpuscular volume (fL)	60.7	73-89
Mean corpuscular hemoglobin (pg)	20.7	24-30
Platelet count (×10⁹/L)	479	150-450
Inflammatory marker		
C-reactive protein (mg/L)	4.06	<5
Electrolytes and renal profile		
Sodium (mmol/L)	136	135-145
Potassium (mmol/L)	3.54	3.5-5.1
Chloride (mmol/L)	94	98-107
Bicarbonate (CO₂) (mmol/L)	15.3	20-28
Creatinine (µmol/L)	29.3	15-37
Glucose (mmol/L)	3.1	3.9-5.8
Venous blood gas		
pH	7.334	7.35-7.45
pCO₂ (mmHg)	23.9	35-45
HCO₃⁻ (mmol/L)	12.4	22-26
Anion gap (mmol/L)	26.2	8-16
Lactate (mmol/L)	2.2	0.5-2.2
Liver and pancreatic profile		
Total protein (g/L)	66.2	60-80
Albumin (g/L)	38.1	35-50
Total bilirubin (µmol/L)	12.4	3-17
ALT (U/L)	34	10-40
AST (U/L)	41	15-50
Alkaline phosphatase (U/L)	298	145-420
Serum amylase (U/L)	<36	28-100

The patient was treated with oral metronidazole 150 mg (10 mg/kg/dose) every eight hours for a planned 10-day course, oral rehydration solution as tolerated, intravenous fluids for dehydration, and oral paracetamol 200 mg every six hours as needed. Within 24 hours of admission, vomiting resolved, oral intake improved, and the child remained active, hemodynamically stable, and well hydrated. Although watery diarrhea persisted at approximately four episodes per day without blood or mucus, abdominal examination remained unremarkable. After 24 hours of hospitalization and clinical observation, he was discharged home to complete the remaining course of oral metronidazole.

At outpatient follow-up on day seven of the prescribed 10-day course of oral metronidazole, the patient was clinically well with complete resolution of vomiting, diarrhea, and abdominal pain. He remained active, playful, and hemodynamically stable and was advised to complete the remaining three days of metronidazole therapy.

Six days after completing the 10-day course of oral metronidazole, the patient presented again with three episodes of non-bilious, non-projectile vomiting and severe upper abdominal pain. According to his parents, he became increasingly irritable, cried persistently, was difficult to console, refused oral intake, and repeatedly pointed to his upper abdomen as the site of pain. There was no associated fever, diarrhea, constipation, or history of abdominal trauma. On examination, he was alert, afebrile, hemodynamically stable, and well hydrated, with age-appropriate vital signs.

Abdominal examination demonstrated tenderness over the upper abdomen without guarding, rebound tenderness, abdominal distension, or rigidity. Bowel sounds were present and normal, with no palpable abdominal masses or organomegaly. Laboratory investigations are summarized in Table 2. These demonstrated leukocytosis with marked neutrophilia and reactive thrombocytosis, markedly elevated pancreatic enzymes (serum amylase 3251 U/L and lipase >1000 U/L), mild hypokalemia, and mild metabolic acidosis. Liver function tests, serum calcium, renal function, random blood glucose, and lipid profile were within normal limits. Repeat stool microscopy showed no *Entamoeba histolytica* cysts or trophozoites. Stool viral antigen testing for rotavirus and adenovirus was negative. Urinalysis was unremarkable, and respiratory viral PCR testing was negative, with no evidence of an active infectious trigger identified at the time of pancreatitis presentation.

**Table 2:** Laboratory investigations during the second admission ALT: alanine aminotransferase, AST: aspartate aminotransferase, pH: potential of hydrogen, pCO₂: partial pressure of carbon dioxide, HCO₃⁻: bicarbonate

Investigation	Result	Pediatric reference interval
Complete blood count		
White blood cell count (×10⁹/L)	21.70	5.0-17.0
Absolute neutrophil count (×10⁹/L)	18.14	1.5-8.5
Absolute lymphocyte count (×10⁹/L)	2.54	2.0-8.0
Hemoglobin (g/dL)	11.7	11.5-13.5
Platelet count (×10⁹/L)	636	150-450
Inflammatory marker		
C-reactive protein (mg/L)	0.6	<5
Electrolytes and renal profile		
Sodium (mmol/L)	139	135-145
Potassium (mmol/L)	3.34	3.5-5.1
Chloride (mmol/L)	104	98-107
Bicarbonate (mmol/L)	20.7	22-28
Anion gap (mmol/L)	14.3	8-16
Creatinine (µmol/L)	27.8	15-37
Random blood glucose (mmol/L)	7.2	3.9-7.8
Liver profile		
Total protein (g/L)	65.6	60-80
Albumin (g/L)	37.4	35-50
Total bilirubin (µmol/L)	8.1	3-17
ALT (U/L)	37	10-40
AST (U/L)	34	15-50
Alkaline phosphatase (U/L)	232	145-420
Pancreatic enzymes		
Serum amylase (U/L)	3251	28-100
Serum lipase (U/L)	>1000	13-60
Minerals and lipid profile		
Calcium (mmol/L)	2.16	2.15-2.65
Triglycerides (mmol/L)	0.65	<1.7
Total cholesterol (mmol/L)	4.35	<5.2
Venous blood gas		
pH	7.338	7.35-7.45
pCO₂ (mmHg)	37.3	35-45
HCO₃⁻ (mmol/L)	19.6	22-26

**Figure 1:**
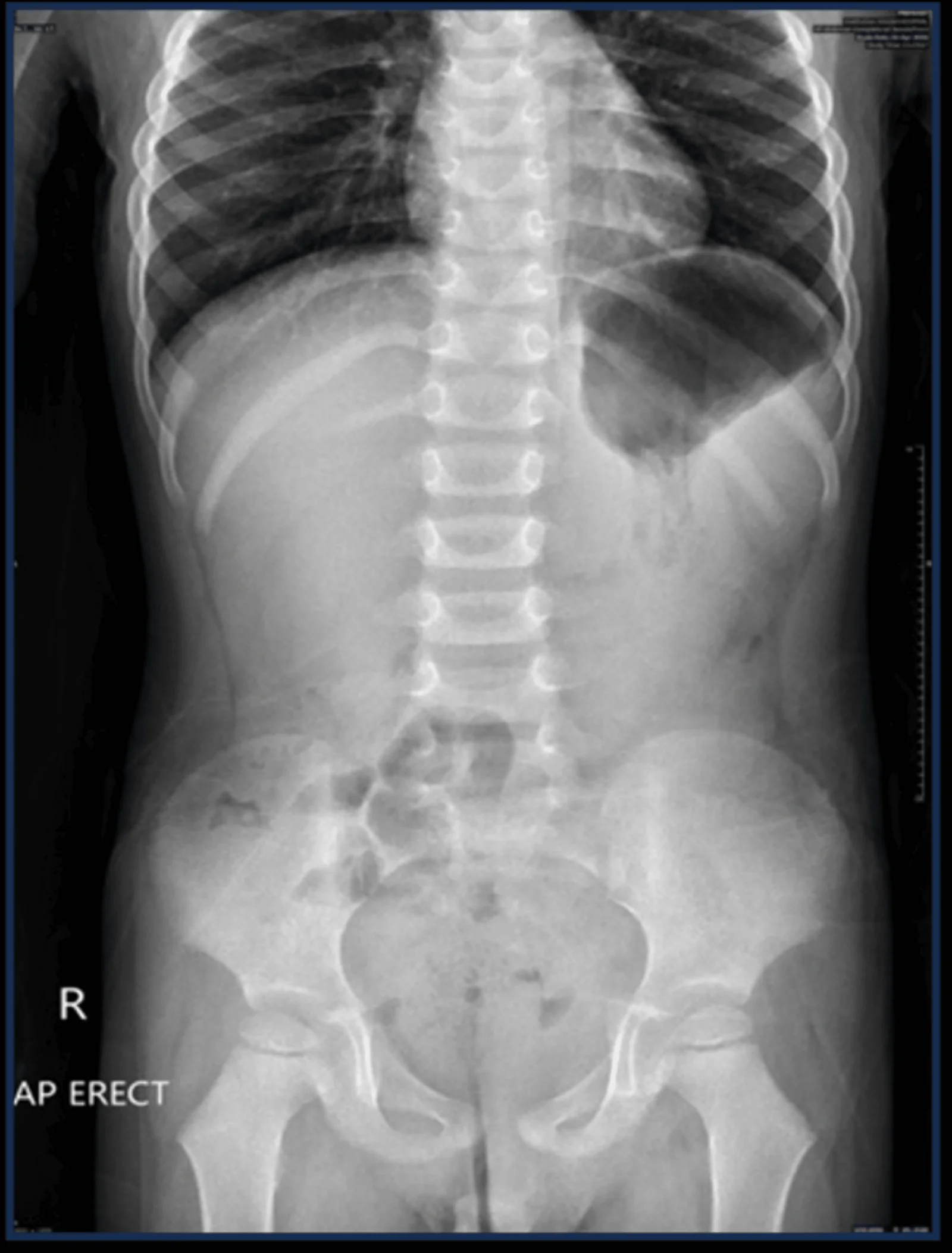
Erect anteroposterior abdominal X-ray demonstrating a normal bowel gas pattern without abnormal air-fluid levels, free intraperitoneal air, or abnormal calcifications

An erect abdominal radiograph (anteroposterior view) was obtained during the initial evaluation and demonstrated no evidence of bowel obstruction or perforation (Figure 1).

**Figure 2:**
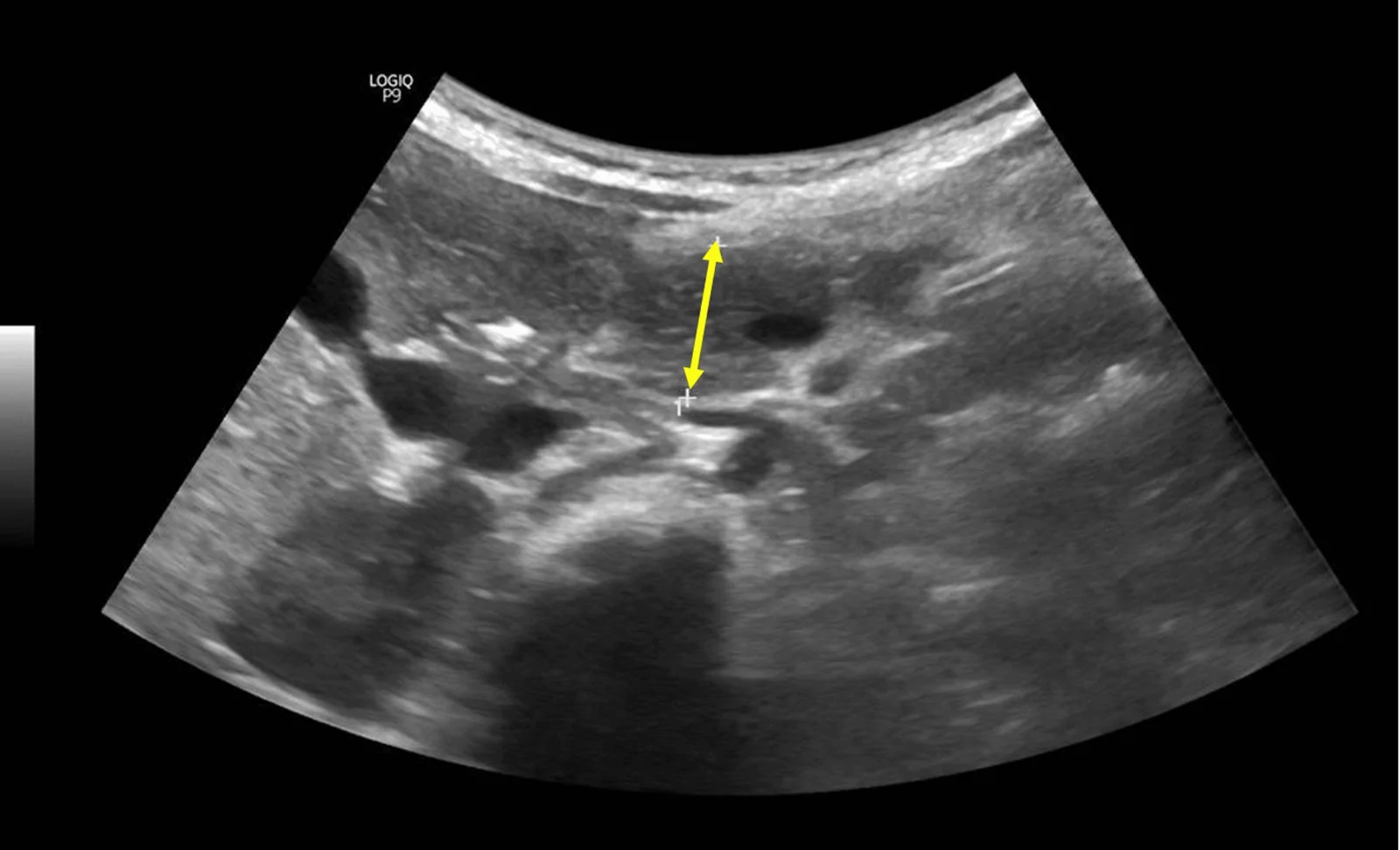
Transverse abdominal ultrasonography showing the visualized portion of the pancreas appearing diffusely enlarged and hypoechoic as illustrated with the yellow arrow

Abdominal ultrasonography demonstrated a mildly enlarged pancreas with mild edematous changes, although visualization was partially limited by overlying bowel gas (Figure 2). No definite peripancreatic fluid collections were identified. A small amount of ascites was noted in the perihepatic and pelvic regions. The gallbladder was normal, with no evidence of gallstones or biliary sludge, and the common bile duct was of normal caliber. Liver, spleen, portal vein, and kidneys were unremarkable. Marked gaseous distension of the colon was also noted. Overall, the ultrasonographic findings were suggestive of AP and should be interpreted in conjunction with the clinical and laboratory findings.

The diagnosis of AP was established according to the INSPPIRE criteria (1), with all three diagnostic features present: characteristic upper abdominal pain, serum amylase and lipase levels exceeding three times the upper limit of normal, and supportive ultrasonographic findings of pancreatic enlargement and edematous changes.

The patient was admitted under pediatric care for management of AP and was evaluated by the pediatric gastroenterology and pediatric surgery teams. Given the absence of surgical indications, no operative intervention was required. Conservative management was initiated, including bowel rest (nil per os), intravenous fluids at 1.5 times maintenance requirements, and analgesia with paracetamol.

During the first 24 hours of hospitalization, the patient remained afebrile and hemodynamically stable. Vomiting resolved completely, and abdominal pain gradually improved. Serial biochemical monitoring demonstrated a marked decline in serum amylase from 3251 IU/L to 119 IU/L and serum lipase from >1000 IU/L to 55 IU/L. The patient remained nil per os and continued to receive intravenous fluids. After 48 hours, oral fluids and a soft diet were gradually reintroduced and were well tolerated without recurrence of abdominal pain or vomiting.

Prior to discharge, repeated abdominal ultrasonography demonstrated interval improvement, with normalization of pancreatic size and resolution of the previously noted ascites. The patient remained clinically well, with adequate oral intake and complete resolution of symptoms, and was discharged home in a stable condition.

The clinical course, including the initiation and completion of metronidazole therapy, subsequent onset of pancreatitis symptoms, diagnostic findings, and clinical recovery, is summarized chronologically in Table 3.

**Table 3:** Clinical timeline of metronidazole exposure and development of AP US: ultrasonography, IV: intravenous, AP: acute pancreatitis

Time point	Clinical events	Investigations	Management/outcome
Day 0	Presented with watery diarrhea, vomiting, and mild generalized abdominal pain	Stool microscopy: *Entamoeba histolytica* cysts detected; rotavirus/adenovirus negative; baseline amylase normal (<36 IU/L)	Diagnosed with presumed amoebic gastroenteritis; started metronidazole 150 mg (10 mg/kg/dose) every 8 hours
Days 1-7	Clinical improvement; vomiting and abdominal pain resolved	-	Continued metronidazole
Day 7	Outpatient follow-up	No evidence of ongoing gastrointestinal symptoms	Advised to complete remaining course
Day 10	Completed 10-day metronidazole course	-	Drug discontinued
Day 16 (6 days after completion)	Re-presented with severe upper abdominal pain, vomiting, irritability, refusal of intake	Amylase 3251 IU/L; lipase >1000 IU/L; US: enlarged hypoechoic pancreas with edema	Diagnosed with AP
Days 16-18	Clinical improvement	Amylase decreased to 119 IU/L; lipase decreased to 55 IU/L; repeat US showed resolution	Supportive management; IV fluids, analgesia, gradual reintroduction of feeding
Discharge	Symptoms resolved	-	Discharged home clinically stable

## Discussion

Metronidazole is a nitroimidazole antimicrobial agent widely used in children and adults to treat anaerobic bacterial and protozoal infections, including amoebiasis caused by *Entamoeba histolytica*. It is generally well tolerated, with gastrointestinal upset, metallic taste, headache, and peripheral neuropathy representing the most frequently reported adverse effects. AP is a rare but recognized adverse drug reaction (ADR) and should be considered in patients presenting with persistent abdominal pain and vomiting during or shortly after metronidazole therapy [5].

DIP accounts for fewer than 5% of all cases of AP, although its true incidence is likely underestimated owing to underrecognition and the absence of a definitive diagnostic test [4]. Consequently, DIP remains a diagnosis of exclusion and relies on a compatible temporal relationship between drug exposure and symptom onset, exclusion of more common etiologies, clinical improvement following withdrawal of the suspected medication, and recurrence upon rechallenge when available [2]. Rechallenge, however, is generally considered unethical in suspected serious ADRs and was therefore not performed in our patient. Applying the Naranjo Adverse Drug Reaction Probability Scale yielded a score consistent with a probable ADR, supporting metronidazole as the most likely precipitating factor.

The diagnosis of AP in our patient fulfilled the INSPPIRE criteria [1], with characteristic abdominal pain manifested by persistent irritability, inconsolable crying, refusal of oral intake, and localization of pain to the upper abdomen, together with serum amylase and lipase levels exceeding three times the upper limit of normal and ultrasonographic findings consistent with AP. In young children, behavioral changes frequently serve as substitutes for verbal localization of pain and should be carefully considered during clinical assessment.

Establishing causality in this case required careful exclusion of alternative etiologies. Although *Entamoeba histolytica* has rarely been implicated in AP, this typically occurs in the setting of severe invasive disease or hepatobiliary involvement. Our patient had an uncomplicated episode of amoebic gastroenteritis that resolved completely following treatment. At the time of re-presentation with pancreatitis, repeat stool microscopy showed no *Entamoeba histolytica* cysts or trophozoites; respiratory viral PCR testing was negative; urinalysis was unremarkable; and there was no clinical evidence of ongoing infection. Furthermore, liver function tests, bilirubin, alkaline phosphatase, serum calcium, triglycerides, renal function, and abdominal ultrasonography showed no evidence of biliary disease, metabolic abnormalities, congenital anomalies, trauma, or systemic illness. Baseline serum amylase obtained during the initial admission for gastroenteritis was within the normal pediatric reference range (<36 U/L), further supporting that pancreatic injury developed only after metronidazole exposure.

A population-based case-control study by Nørgaard et al. demonstrated an approximately threefold increased risk of AP among metronidazole users, providing epidemiological support for this association [7]. Although the exact mechanism of metronidazole-induced pancreatitis remains uncertain, several hypotheses have been proposed. Reactive oxygen species generated during metronidazole metabolism may induce oxidative injury to pancreatic acinar cells, while idiosyncratic immune-mediated mechanisms and individual genetic susceptibility may also contribute [8-10].

As summarized in Table 4, previously reported cases of metronidazole-induced AP have almost exclusively involved adults, predominantly women, with symptom onset occurring from within 24 hours to eight days after initiation of therapy. Several reports documented recurrence following re-exposure, providing further evidence for causality. To our knowledge, pediatric cases remain exceedingly rare, and our patient represents one of the youngest reported cases of suspected metronidazole-associated AP. The six-day interval between completion of metronidazole therapy and symptom onset falls within the latency period described in previous reports.

**Table 4:** Previously reported cases of metronidazole-associated AP and comparison with the present case AP: acute pancreatitis

Reference	Gender	Age (years)	Indication for metronidazole	Episode	Interval between metronidazole exposure and pancreatitis onset (days)
Feola and Thornton (2002) [11]	Female	22	Unspecified vaginosis	1st episode	<1
Feola and Thornton (2002) [11]	Female	22	Unspecified vaginosis	2nd episode	1
Feola and Thornton (2002) [11]	Female	22	Unspecified vaginosis	3rd episode	1
Sura et al. (2000) [9]	Female	23	Bacterial vaginosis	1st episode	8
Plotnick et al. (1985) [12]	Female	29	Postpartum unspecified vaginitis	1st episode	1
Plotnick et al. (1985) [12]	Female	29	Postpartum unspecified vaginitis	2nd episode	37
Corey et al. (1991) [13]	Female	49	Trichomoniasis	1st episode	3–5
Corey et al. (1991) [13]	Female	49	Trichomoniasis	2nd episode	<1
Nigwekar and Casey (2004) [5]	Female	46	Bacterial vaginosis	1st episode	8
Nigwekar and Casey (2004) [5]	Female	46	Bacterial vaginosis	2nd episode	8
Sanford et al. (1988) [14]	Female	63	Crohn’s disease	Single episode	7
O'Halloran et al. (2010) [15]	Female	25	Endodontic abscess	1st episode	2
O'Halloran et al. (2010) [15]	Female	25	Endodontic abscess	2nd episode	1
Celifarco et al. (1989) [16]	Female	61	Aspiration pneumonia	Single episode	4
Our case	Male	3	Entamoeba histolytica	Single episode	6

There are no universally accepted diagnostic criteria for DIP; therefore, the diagnosis remains one of exclusion. It is primarily based on a compatible temporal relationship between drug exposure and symptom onset, exclusion of more common etiologies, improvement after withdrawal of the suspected agent when assessable, and recurrence following rechallenge when available. In the present case, the patient developed AP six days after completing metronidazole therapy. At the same time, alternative causes, including biliary disease, hypertriglyceridemia, hypercalcemia, trauma, ongoing infection, and systemic illness, were excluded through clinical assessment, laboratory investigations, and abdominal ultrasonography. Although improvement following drug withdrawal could not be assessed because the metronidazole course had already been completed before symptom onset, rechallenge was not performed because of ethical considerations. Application of the Naranjo Adverse Drug Reaction Probability Scale [17] yielded a score of 6 (Table 5), indicating a probable ADR. This assessment further supports metronidazole as the most likely precipitating factor in the current case.

**Table 5:** Naranjo Adverse Drug Reaction Probability Scale assessment for suspected metronidazole-induced AP ADR: adverse drug reaction, AP: acute pancreatitis

Question	Response	Score
Previous conclusive reports of this reaction?	Yes	+1
Did the event occur after drug administration?	Yes	+2
Did the reaction improve after drug discontinuation?	Not assessable	0
Did the reaction recur after re-administration?	Not performed	0
Are there alternative causes that could have caused the reaction?	No	+2
Did the reaction recur with placebo?	Not applicable	0
Was the drug detected at toxic concentrations?	No	0
Was there a dose-response relationship?	Unknown	0
Previous similar reaction?	No	0
Was the adverse event confirmed by objective evidence?	Yes	+1
Total score	-	6 (probable ADR)

## Conclusions

This case describes a rare presentation of suspected metronidazole-associated AP in a three-year-old child following treatment for amoebic gastroenteritis. While a definitive causal relationship cannot be established from a single case report, the temporal association between metronidazole exposure and symptom onset, objective confirmation of AP, comprehensive exclusion of alternative etiologies, and a probable causality assessment according to the Naranjo Adverse Drug Reaction Probability Scale support metronidazole as a possible contributing factor in this case. The purpose of reporting this case is not to establish causation but to raise clinical awareness regarding this rare potential ADR and encourage consideration of AP in children who develop persistent vomiting and upper abdominal pain during or shortly after metronidazole exposure. Further pediatric case reports and future studies are required to better characterize the potential association between metronidazole and AP in children.
